# Monitoring Upper Limb Recovery after Cervical Spinal Cord Injury: Insights beyond Assessment Scores

**DOI:** 10.3389/fneur.2016.00142

**Published:** 2016-08-31

**Authors:** Michael Brogioli, Sophie Schneider, Werner L. Popp, Urs Albisser, Anne K. Brust, Inge-Marie Velstra, Roger Gassert, Armin Curt, Michelle L. Starkey

**Affiliations:** ^1^Spinal Cord Injury Center, Balgrist University Hospital, Zurich, Switzerland; ^2^Rehabilitation Engineering Laboratory, Department of Health Sciences and Technology, ETH Zurich, Zurich, Switzerland; ^3^Clinical Trial Unit, Swiss Paraplegic Centre, Nottwil, Switzerland

**Keywords:** spinal cord injury, upper limb, rehabilitation, long-term monitoring, wearable sensors

## Abstract

**Background:**

Preclinical investigations in animal models demonstrate that enhanced upper limb (UL) activity during rehabilitation promotes motor recovery following spinal cord injury (SCI). Despite this, following SCI in humans, no commonly applied training protocols exist, and therefore, activity-based rehabilitative therapies (ABRT) vary in frequency, duration, and intensity. Quantification of UL recovery is limited to subjective questionnaires or scattered measures of muscle function and movement tasks.

**Objective:**

To objectively measure changes in UL activity during acute SCI rehabilitation and to assess the value of wearable sensors as novel measurement tools that are complimentary to standard clinical assessments tools.

**Methods:**

The overall amount of UL activity and kinematics of wheeling were measured longitudinally with wearable sensors in 12 thoracic and 19 cervical acute SCI patients (complete and incomplete). The measurements were performed for up to seven consecutive days, and simultaneously, SCI-specific assessments were made during rehabilitation sessions 1, 3, and 6 months after injury. Changes in UL activity and function over time were analyzed using linear mixed models.

**Results:**

During acute rehabilitation, the overall amount of UL activity and the active distance wheeled significantly increased in tetraplegic patients, but remained constant in paraplegic patients. The same tendency was shown in clinical scores with the exception of those for independence, which showed improvements at the beginning of the rehabilitation period, even in paraplegic subjects. In the later stages of acute rehabilitation, the quantity of UL activity in tetraplegic individuals matched that of their paraplegic counterparts, despite their greater motor impairments. Both subject groups showed higher UL activity during therapy time compared to the time outside of therapy time.

**Conclusion:**

Tracking day-to-day UL activity is necessary to gain insights into the real impact of a patient’s impairments on their UL movements during therapy and during their leisure time. In the future, this novel methodology may be used to reliably control and adjust ABRT and to evaluate the progress of UL rehabilitation in clinical trials.

## Introduction

Cervical spinal cord injury (SCI) results in profound and devastating life changes for the affected individuals due to the loss of arm and hand function (1). Consequently, this function is the one that tetraplegics would most like to regain (2, 3). However, there is currently no effective treatment for SCI (4–6), damaged axons do not repair spontaneously, and regenerative growth is extremely limited, if it happens at all (7). Therefore, the functional recovery that is observed is either due to functional compensation or plastic changes in intact fibers (8). Preclinical data suggest that functional reorganization of the adult mammalian central nervous system (CNS) can be promoted through activity-based rehabilitative therapies (ABRT) (9), which have been shown to improve forelimb function and enhance plastic sprouting of undamaged corticospinal tract fibers in adult rats (10–14).

In clinical research, the influence of UL activity on functional recovery is less clear. This is on the one hand, because there are few studies investigating this issue and on the other hand because the results that do exist are contradictory (15). Typical challenges to such studies are the limited sample size due to low incidence of SCI, frequent subject dropout, and poor adherence due to a high frequency of secondary complications in cervical patients as well as the fact that UL movements are complex because they involve a variety of non-cyclic movements that are difficult to measure objectively (1, 16). The latter may be the reason why no commonly applied training protocols exist. The consequence is that ABRT are highly variable resulting in different protocols in terms of both training characteristics (e.g., frequency, duration, or intensity) and outcome measures used to test their efficacy (16). Additionally, the assessment of UL activity outside of training sessions is often limited to self-reported questionnaires that have been shown to be rather imprecise, overestimating the actual activity of the subject (17). As a consequence, the efficacy of ABRT, which can be evaluated in terms of increased quantity of UL movements, is difficult to assess. This is because functional improvements cannot be associated exclusively with ABRT-induced increases in neuronal activity, as the overall UL activity performed outside therapy sessions cannot be accurately assessed. Therefore, an objective daylong measure of performance is needed to assess the effect of an activity-based increase in neuronal activity on functional recovery and to track the evolution over the inpatient stay.

The use of wearable sensors during SCI rehabilitation could be a feasible solution for measuring total UL activity. Wearable sensors provide objective and continuous measures so that outcomes can be compared between studies (18). In this regard, wearable sensors have been used in the field of SCI research to determine everyday physical activity (19–21). However, as these studies focused exclusively on measuring physical activity rather than assessing functional recovery, they were not performed within standardized time frames and the activity outcomes were not compared with standardized clinical outcomes (19–21). For this reason, in a previous study, we showed the feasibility and validity of sensor-based outcome metrics in measuring UL function and independence during cross-sectional recordings (22). Given the validity and sensitivity of these measures, the purpose of this study was to assess the quantity of upper limb (UL) activity and its changes during acute rehabilitation in a cohort of tetraplegic and paraplegic patients in standardized SCI-specific time frames.

## Materials and Methods

### Subjects

The 31 subjects with SCI (age 47.84, SD: ±17.50 years, range: 20–77 years, ASIA A-D, 12 paraplegic and 19 tetraplegic subjects, 22 males and 9 females) participated in this study. Additional demographic information can be found in Table 1. Participants were recruited from the Swiss Paraplegic Centre in Nottwil, Switzerland, the Balgrist University Hospital in Zurich, Switzerland, and the Rehab Basel in Basel, Switzerland. Acute wheelchair-bound patients with a traumatic SCI were included in this study 1 month (Acute I, 16–40 days, 30 subjects) or 3 months (Acute II, 70–98 days, 31 subjects) after injury according to the time frames of the European Multicenter Study about SCI (EMSCI^1^). Patients with a neurological disease other than SCI as well as those with an orthopedic or rheumatologic disease were excluded from this study. Measurements were performed at 1, 3, and 6 months (Acute III, 150–186 days, 27 subjects) after injury within the EMSCI time-windows. All patients were measured in at least two different time windows, and 26 of these were measured in all three time windows. The study was approved by the ethical committees of the cantons of Zurich, Lucerne, and Basel. All participants gave their written informed consent in accordance with the Declaration of Helsinki.

**Table 1:** **Demographic characteristics of the 31 spinal cord injured subjects included in the study**.

Subject	Age	Gender	Neurological level of injury	ASIA Impairment Scale
1	32	Male	C3	D
2	71	Male	C3	D
3	60	Male	C3	D
4	31	Male	C4	A
5	53	Female	C4	D
6	22	Male	C4	D
7	37	Male	C4	D
8	33	Male	C5	A
9	25	Male	C5	A
10	63	Female	C5	D
11	53	Male	C5	D
12	49	Male	C5	D
13	60	Female	C5	D
14	73	Female	C5	D
15	75	Male	C5	D
16	55	Female	C6	D
17	38	Male	C7	A
18	20	Male	C7	B
19	60	Male	C7	D
20	53	Female	T5	B
21	32	Male	T6	D
22	28	Male	T8	A
23	49	Female	T8	C
24	44	Female	T10	A
25	58	Male	T10	A
26	77	Male	T10	A
27	65	Male	T11	C
28	29	Male	T11	D
29	74	Male	T12	D
30	25	Female	L2	A
31	39	Male	L2	D

### Clinical Assessments

Neurological impairment was assessed with the International Standards for Neurological Classification of Spinal Cord Injury (ISNCSCI) protocol (23). This protocol classifies the neurological level of injury (NLI) and the extent of lesion by determining the most caudal intact myotome or sensory dermatome. Observed NLI levels range from C2 (cervical spinal cord segment) to S4–5 (sacral spinal cord segment). Cervical (tetraplegic; above T2) and thoracic (paraplegic; T2 and below) patients were grouped according to the NLI value at 3 months after injury, as this information was available for all patients. This information was used to define the two investigated groups as explained in Section “Statistical Analysis.” The extent of lesion was assessed according to the ASIA Impairment Scale (AIS).

Motor function of the UL was assessed using the motor domain of the Graded and Redefined Assessment of Strength, Sensibility and Prehension (GRASSP) (24, 25) that assesses the function of 10 UL muscles on both arms with the manual muscle testing (MMT). The scores range from 0 to 50 per arm, and the scores of both arms were summed together. In a previous study, we showed that proximal motor scores of the GRASSP are strongly related to overall UL activity in acute inpatients (22); therefore, distal muscle scores were omitted from the analysis, resulting in a proximal score range from 0 to 20 per arm. Strength tests with a hand-held dynamometer (HHD) of four key groups of UL muscles were performed: elbow flexors (biceps brachii, brachialis, and brachioradialis), elbow extensors (triceps brachii), shoulder flexors (deltoid anterior part, pectoralis major upper and middle part), and extensors (latissimus dorsi and teres major) (26). This assessment tool was chosen in order to obtain a more sensitive measure of strength values from M3 to M5 (27). Hand grip strength was measured with a hand dynamometer (28).

Independence in self-care was assessed with the self-care subdomain of the Spinal Cord Independence Measure (SCIM) (29), resulting in a score range from 0 to 20.

### Data Collection and Measurement Procedure

**Figure 1:**
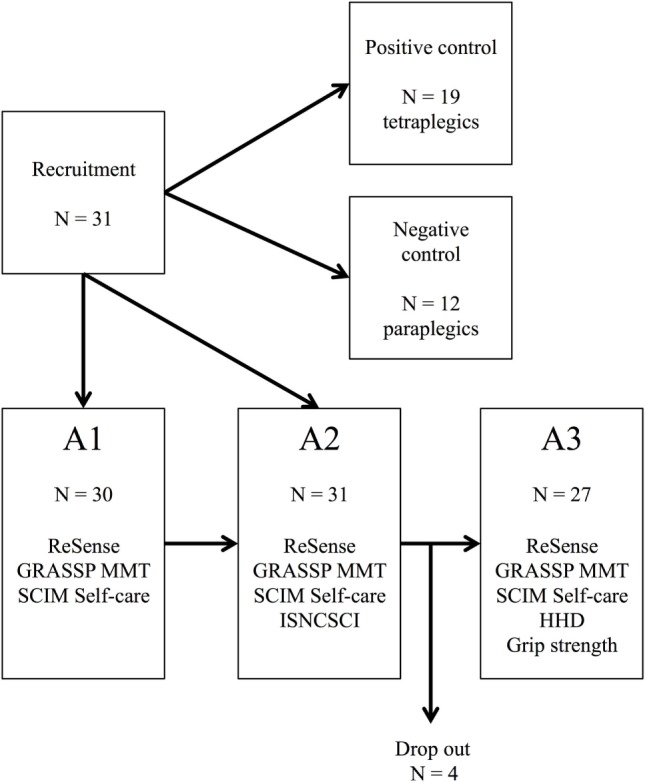
**Flow diagram depicting the study groups and the measurement performed in each time frame**. Stage A1: 1 month after injury; Stage A2: 3 months after injury; Stage A3: 6 months after injury; GRASSP, Graded and Redefined Assessment of Strength, Sensibility and Prehension; SCIM, Spinal Cord Independence Measure; HHD, hand-held dynamometer; N, sample size; MMT, Manual Muscle Testing; ISNCSCI, International Standards for Neurological Classification of Spinal Cord Injury.

Patients were assessed three times during primary inpatient rehabilitation (Figure 1). Each time frame consisted of three weekdays of wearable sensor recordings in conjunction with clinical assessments. The wearable sensor used in this study was the ReSense (30), an inertial measurement unit that records 3D acceleration, 3D angular velocity, 3D magnetic field strength, and barometric pressure for at least 24 h at a time. If only 3D acceleration is measured, then the battery life lasts for over 2 weeks. Signals coming from the magnetometer and the barometric pressure sensor were disregarded for the purposes of this study. For the recordings, patients were fitted with three ReSense modules, one on each wrist and one on the right wheel of the wheelchair. The wheel module remained fixed on the wheel for up to 7 days, recording wheeling kinematics. More details about the ReSense set-up are presented elsewhere (31, 32). Patients were not asked to perform any specific activity, but they were free to behave as they wanted following their daily inpatient schedule. ReSense had to be removed only during bathing or any activity involving long-term contact with water. GRASSP examinations were performed by trained research staff consisting of movement scientists, occupational therapists, and physiotherapists. The SCIM questionnaire and the ISNCSCI protocols were rated by clinicians who were independent to the study.

### Data Analysis

ReSense data were transferred post-recording from the internal SD-card *via* a custom-designed base station to a PC and were analyzed offline using MATLAB R2013a (MathWorks, Natick, MA, USA). A cubic spline interpolation function was used to resample the data at 50 Hz enabling the synchronization of recordings from different sensor modules. Visual inspection was performed in order to ensure that the data were genuine, removing data recorded during sleep phases and phases when the sensors were taken off prior to the analysis.

### Sensor-Based Outcome Measures

In order to track changes in UL activity, we used sensor-based metrics [overall activity counts (ACs), distance wheeled, peak wheeling velocity, and limb-use laterality index] that allow a comprehensive evaluation of UL recovery as they have been shown to be closely related to UL motor function and independence in an acute cross-sectional study (22).

Activity count was used as a measure of overall UL activity. In order to calculate this metric, the acceleration signal is processed with a second-order Butterworth high-pass filter with a cut-off frequency of 0.25 Hz. Subsequently, the magnitude of the filtered signal was integrated over an epoch of 1 min, resulting in an output in counts per min. The counts of the right and left limb were summed together and normalized by time.

Limb-use laterality refers to the dominance in the usage of one UL over the other during day-to-day activities. Limb-use laterality was assessed with the ReSense Assessment of Laterality (RSAL) and is scored from 0 to infinite, where the higher, the value the more pronounced the limb-use laterality (31, 33). Lateralized patients were defined here as patients with limb-use laterality values above 2 SDs from the mean of paraplegic subjects at 1 month after injury (*Z*-score = 2).

Distance actively wheeled and peak velocity was calculated over an extended amount of time of up to 7 days (34) with an algorithm previously developed by our group (32). In short, the ReSense Wheeling-Algorithm (RSWA, set-up II.a and III.b) reliably discriminates active (self-propelled) and passive (attendant-propelled) wheeling estimating speed (meters per second) and distance (in meters). In this way, active distance wheeled and peak wheeling velocity can be reliably measured. Peak velocity was computed using the 90th percentile in order to obtain a more robust metric against outliers in peak velocity.

### UL Activity Categories

We split up overall AC into two distinct activity categories because overall AC during the whole day is a generic measure. In agreement with our previous study (31), these two categories were distinguished based on the output of the RSWA (set-up II.a). The category “self-propulsion AC” included all upper extremity movements performed while the subject actively propelled the wheelchair, whereas the category “ADL AC” included all upper extremity movements that occurred during any other day-to-day activities excluding self-propulsion. In addition, the difference between AC performed during therapies and AC performed outside therapy sessions was evaluated by splitting a day into therapy time (from 9:00 a.m. to 5:00 p.m.) and leisure time (time outside 9:00 a.m. to 5:00 p.m. excluding sleep).

### Statistical Analysis

The statistical analysis was performed using IBM SPSS Statistics version 19 (IBM, Armonk, NY, USA). Figures were prepared using the ggplot2 library for R (The R project for Statistical Computing, R Core Team^2^). Two analyses were performed: a longitudinal analysis over all time frames (analysis of changes) and a cross-sectional analysis at 6 months after injury (analysis of the differences between groups). The measured subjects were divided into two groups according to the NLI value at 3 months after injury: a control group of paraplegic subjects in which no changes in UL activity are expected and a group of tetraplegic subjects in which improvements in UL activity are expected.

#### Sample Size

We recruited 31 SCI patients who were heterogeneous in terms of their impairments and in how they mobilize. For these reasons, the number of subjects included in different analyses varies depending on the aim of the analysis. If not otherwise stated, the sample size is 31 patients (19 tetraplegic patients and 12 paraplegic patients) for the longitudinal analysis and the cross-sectional analysis at stage A2, 30 patients (18 tetraplegics patients and 12 paraplegic patients) for the cross-sectional analysis at stage A1, and 27 patients (16 tetraplegic patients and 11 paraplegic patients) for the cross-sectional analysis at stage A3 (Figure 1). The sample size is stated in parenthesis in case of smaller sample sizes due to not tested items in the clinical assessment of some individuals.

#### Longitudinal Analysis

Data have been analyzed with a linear mixed model (LMM) due to inconsistent sample sizes across stages. The repeated-measures dataset was considered to be a two-level type, in which the second level represents the patient and therefore covariates measured at this level represent between-subject variation. The first level represents the repeated measurements made on each patient and therefore within-subject variation. To analyze each dependent variable, six statistical models were built: overall AC, active distance wheeled, peak velocity, limb-use laterality, GRASSP MMT proximal, and SCIM self-care. For all models, subjects and intercept were included as random factors. Covariates, main effects, and interaction effects were included as fixed effects. The following fixed effects were used to set up the statistical models: age and gender were treated as covariates. The main effect time was chosen as repeated measurement, and its residual covariance matrix was set to uncorrelated and estimated with the restricted maximum likelihood. In order to test interaction effects, grouping variables were added to the model and defined as the category paresis (0 = paraplegic patient, 1 = tetraplegic patient) and the category limb-use laterality (0 = no UL lateralization, 1 = UL lateralization, limb-use laterality model only). The interaction time × paresis was added to all models. The interaction time × limb-use laterality was added to the limb-use laterality model.

The predicted means of each category (e.g., paraplegic patients) were computed for each time frame using the fitted model. In order to discover whether the mean of a group was equal over all time-windows, a univariate test was performed. If the means were different, pairwise comparisons were employed to identify significant differences between specific time frames. For this purpose, the alpha level was adjusted for multiple comparisons using Bonferroni correction. All *p*-values reported are corrected for multiple comparisons.

#### Cross-sectional Analysis

The comparison between paraplegic and tetraplegic groups was performed either with an independent sample *t*-test, in the case that the data were normally distributed, or with the non-parametric Mann–Whitney *U* test in the case of non-normally distributed data. Normality was checked with the Shapiro–Wilk test of Normality (35). Normality was not met for the values of limb-use laterality and all the scores of the clinical assessments. In case of multiple means comparisons (i.e., more than two), a one-way analysis of variance (one-way ANOVA) with Bonferroni *post hoc* test was performed.

A Spearman’s rank-order correlation coefficient was used to inspect the associations between sensor metrics and assessment scores.

For all statistical tests, the statistical significance level α was set at 0.05.

## Results

### Changes in Sensor Metrics

**Figure 2:**
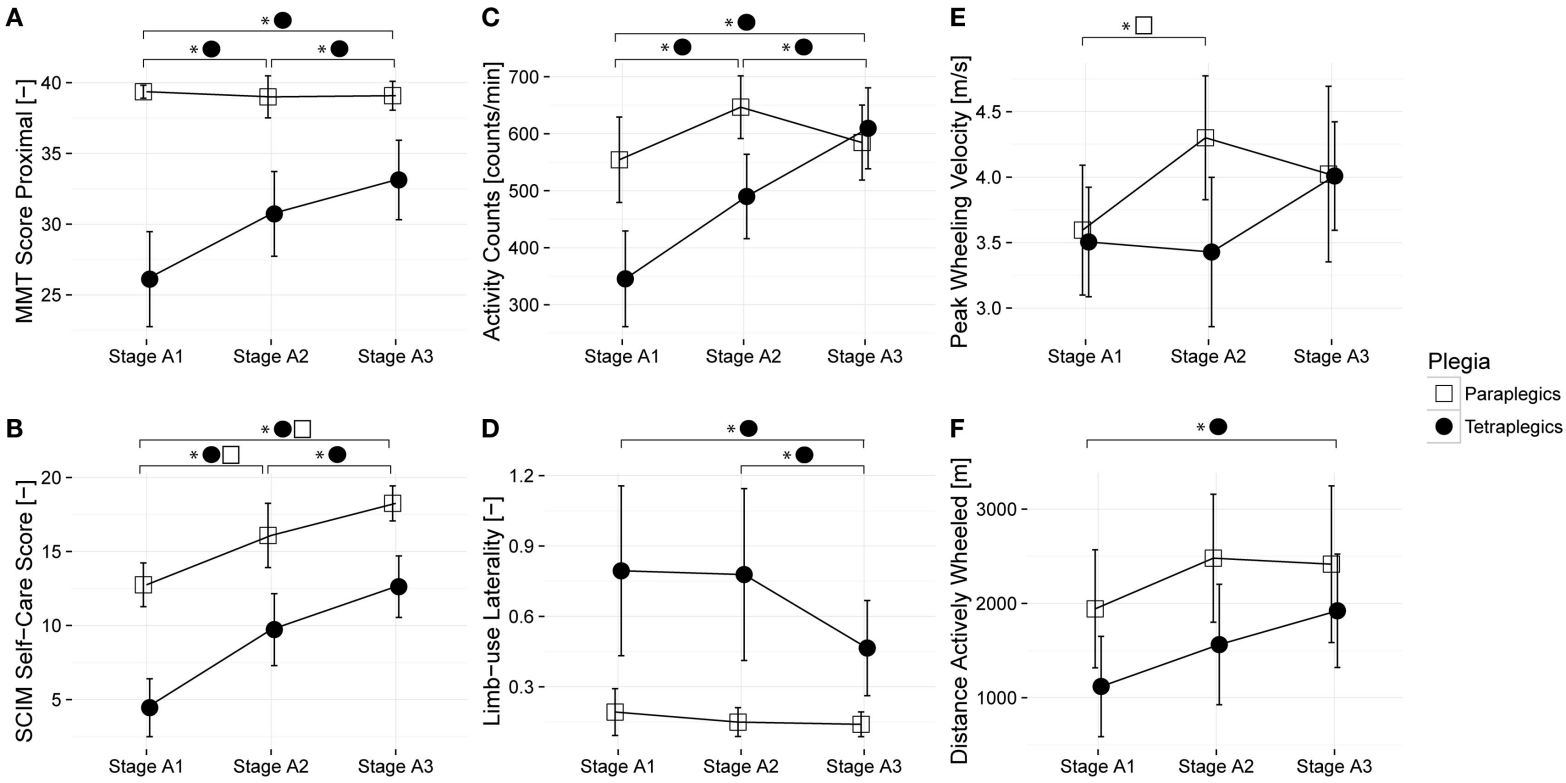
**Changes in sensor-based and clinical measures over time among a group of paraplegic and tetraplegic patients**. Lines represent the means; error bars represent the 95% confidence interval. Paraplegic patients are displayed with empty squares, whereas tetraplegic patients are displayed with full circles. **(A,B)** illustrate the changes in clinical scores during rehabilitation, **(C–F)** changes in sensor-based metrics. Proximal muscle strength was assessed with the manual muscle testing (MMT); independence in self-care was assessed with the Spinal Cord Independence Measure (SCIM). Stage A1 – 1 month after injury; Stage A2 – 3 months after injury; and Stage A3 – 6 months after injury.

The aim of this study was to examine changes in sensor-based measures across time among a group of paraplegic and tetraplegic subjects (Figure 2). For this purpose, changes in six dependent variables (four sensor metrics and two clinical assessment measures) were analyzed using LMM. The six dependent variables were overall AC, distance wheeled actively, peak wheeling velocity, limb-use laterality, GRASSP MMT proximal, and SCIM self-care. Results of pairwise comparisons of the estimated marginal means over the three time frames for paraplegic and tetraplegic patients are summarized in Table 2.

**Table 2:** **Summary of changes in overall upper limb activity, distance wheeled per day, peak velocity, and limb-use laterality**.

Group	Time	GRASSP MMT (scores)	SCIM self-care (scores)	Overall activity (counts/min)	Distance (m/day)	Peak velocity (m/s)	Laterality index	Group	Laterality index
**Paraplegics**	1 month	39.464 (2.056)	12.706 (1.313)	552.617 (57.727)	1889.160 (376.139)	3.531 (0.259)	0.192 (0.233)	Non-lateralized	0.159 (0.122)
	3 months	39.100 (1.904)	16.039 (1.653)	644.973 (47.763)	2549.482 (407.526)	4.306 (0.277)	0.149 (0.213)		0.226 (0.138)
	6 months	39.099 (1.615)	18.206 (1.244)	589.342 (47.122)	2261.312 (473.384)	4.185 (0.318)	0.136 (0.111)		0.203 (0.093)
	Significant pairwise comparisons (*p* < 0.05^a^)	ns	*t*_1_–*t*_2_, *t*_1_–t_3_	ns	ns	*t*_1_–*t*_2_	ns		ns
**Tetraplegics**	1 month	25.715 (1.619)	4.361 (1.058)	331.316 (46.531)	1045.859 (320.275)	3.492 (0.263)	0.896 (0.186)	Lateralized	1.344 (0.173)
	3 months	31.046 (1.503)	9.791 (1.326)	495.693 (38.376)	1677.737 (360.708)	3.374 (0.263)	0.742 (0.170)		0.890 (0.194)
	6 months	33.853 (1.311)	13.003 (1.037)	627.111 (38.337)	2286.398 (424.393)	4.120 (0.307)	0.432 (0.089)		0.453 (0.130)
	Significant pairwise comparisons (*p* < 0.05^a^)	*t*_1_–*t*_2_, *t*_1_–*t*_3_, *t*_2_–*t*_3_	*t*_1_–*t*_2_, *t*_1_–*t*_3_, *t*_2_–*t*_3_	*t*_1_–*t*_2_, *t*_1_–*t*_3_, *t*_2_–*t*_3_	*t*_1_–*t*_3_	ns	*t*_1_–*t*_3_, *t*_2_–*t*_3_		*t*_1_–*t*_2_, *t*_1_–*t*_3_, *t*_2_–*t*_3_

*^a^Bonferroni corrected; ns, not significant; t_1_, 1 month; t_2_, 3 months; t_3_, 6 months. Results are displayed as estimates ± SEs*.

**Figure 3:**
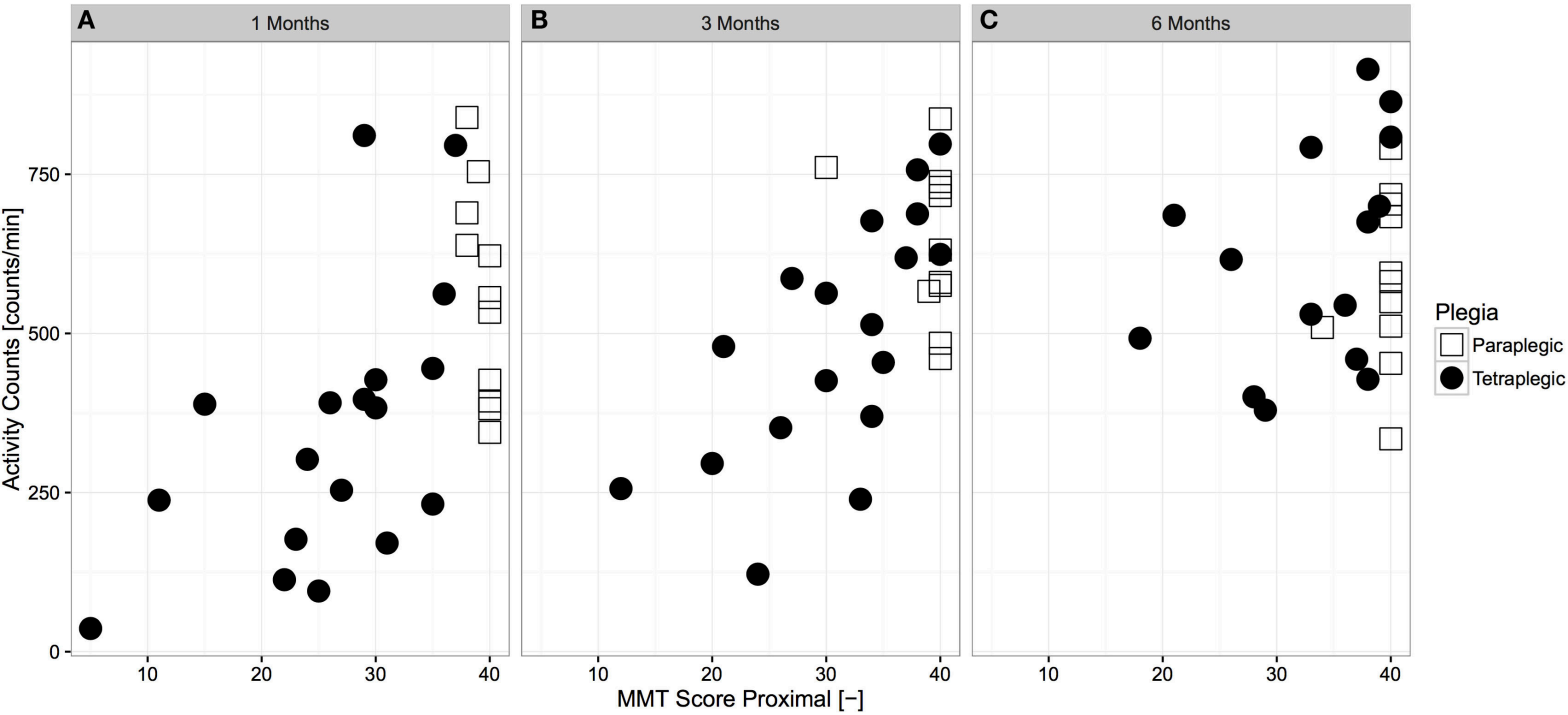
**Cross-sectional relationship between proximal muscle function and overall upper limb activity across time**. Paraplegic patients are displayed with empty squares, whereas tetraplegic patients are displayed with full circles. The relationship at 1 month **(A)** and 3 months **(B)** after injury was strong and significant (*N* = 29 and *N* = 31, *p* < 0.01, *r* = 0.562 and *r* = 0.605, Spearman correlation, respectively), whereas it was not significant at 6 months **(C)** after injury (*N* = 27, *p* = 0.178, *r* = 0.273, Spearman correlation). MMT, manual muscle testing.

The relationship between overall AC and proximal muscle function was analyzed for each time frame (Figure 3). Overall AC and proximal muscle function were strongly related at 1 month (*p* < 0.01, *r* = 0.562, *N* = 29, Spearman correlation) and 3 months (*p* < 0.01, *r* = 0.605, *N* = 29, Spearman correlation) after injury, though the relationship was not significant at 6 months after injury (*p* = 0.178, *r* = 0.273, Spearman correlation).

### Changes in Limb-Use Laterality

As shown in Table 2, pathologically increased limb-use laterality significantly decreased in tetraplegic subjects, whereas, as expected, it remained unchanged throughout the study in paraplegic subjects. A Mann–Whitney test revealed that limb-use laterality of tetraplegic subjects was significantly more pronounced over the course of acute care 1 and 3 months after injury (mean rank = 18.50, 18.44) than for paraplegic subjects (mean rank = 11.00 and 11.08; *U* = 54 and 55; *z* = −2.286 and −2.244; *p* < 0.05 and *p* < 0.05). Limb-use laterality of tetraplegic subjects seemed to recover at the end of the acute rehabilitation at 6 months after injury (mean rank = 16.25) as at this time, it was not significantly different from the paraplegic subjects (mean rank = 10.73, *U* = 52, *Z* = −1.776, *p* = 0.07). In contrast to the 75th percentile (0.237 for paraplegic subjects and 1.110 for tetraplegic subjects), the 25th percentile (0.038 for paraplegics and 0.129 for tetraplegic) of the laterality index at 1 month after injury was comparable between paraplegic and tetraplegic subjects, meaning that some tetraplegic subjects showed the same limb-use laterality as paraplegic subjects. For this reason, limb-use laterality was further analyzed for a cohort of lateralized subjects. In this case, lateralized subjects were defined as those subjects whose laterality values at 1 month were more than 2 SDs of the mean of paraplegic subjects (i.e., laterality index above 0.6127). Nine subjects (eight tetraplegic subjects and one paraplegic subject) showed lateralization. Limb-use laterality significantly decreased in these lateralized subjects (Table 2) but remained significantly different from their non-lateralized counterparts in all time windows, meaning that lateralized subjects recover some limb-use symmetry but remain impaired in terms of laterality (mean rank no lateralization = 10.50, 11.79, and 11.18; mean rank lateralization = 25.50, 21.10, and 17.89; *U* = 0, 34, and 37; *z* = −4.399, −2.799, and −2.129; *p* < 0.01, *p* < 0.01, and *p* < 0.05).

### Group Differences at 6 Months

**Figure 4:**
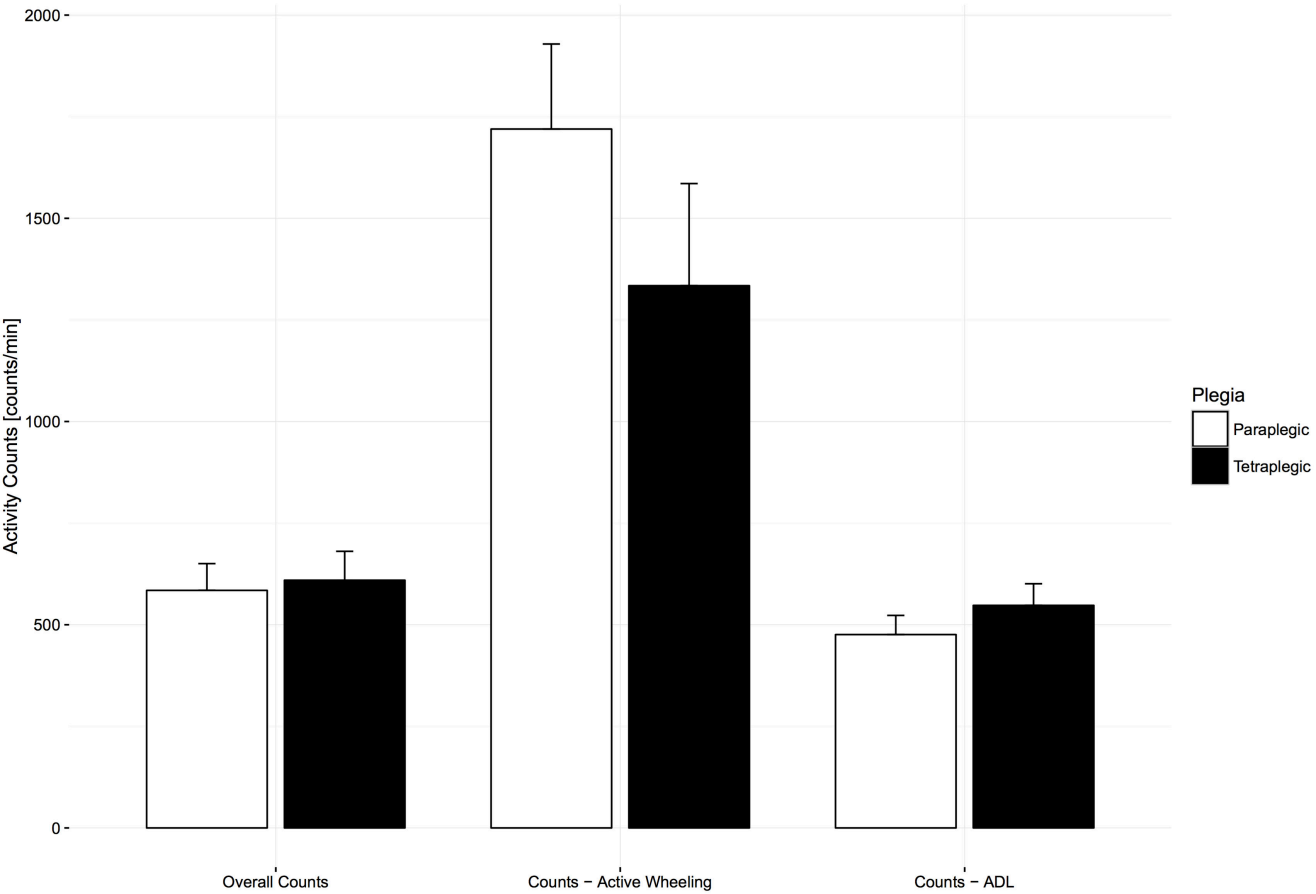
**Comparison of activity count (AC) categories between paraplegic and tetraplegic patients 6 months after injury**. Bars represent the means; error bars represent the 95% confidence interval. Paraplegic patients are displayed in white, whereas tetraplegic patients are displayed in black. Differences are not statistically significant. ADL, activities of daily living.

To determine if there was a discrepancy in UL activity between paraplegic and tetraplegic subjects at 6 months after injury, comparisons between group means were performed for different UL activity categories (overall AC, ADL AC, and self-propulsion AC). An independent samples *t*-test revealed that overall AC [584.50 ± 132.83 counts/min for paraplegic and 609.60 ± 172.70 counts/min for tetraplegic, *t*(25) = −0.43, *p* = 0.67] and ADL AC [475.79 ± 85.93 counts/min for 9 paraplegic and 547.60 ± 112.17 counts/min for 12 tetraplegic, *t*(19) = −1.66, *p* = 0.11] were not significantly different between the two groups (Figure 4). Finally, 27 paraplegic and tetraplegic subjects had higher counts during therapy times (618.28 ± 153.80 and 695.97 ± 193.99 counts/min) compared to leisure time (536.02 ± 122.16 and 514.47 ± 180.92 counts/min). The increase in counts from leisure time to therapy time was slightly more significant in 16 tetraplegics [181.49 (95% CI, 99.04–263.95) counts/min, *t*(15) = 4.692, *p* < 0.01] compared to 11 paraplegics [82.26 (95% CI, 1.19–163.33) counts/min, *t*(10) = 2.261, *p* < 0.05].

**Figure 5:**
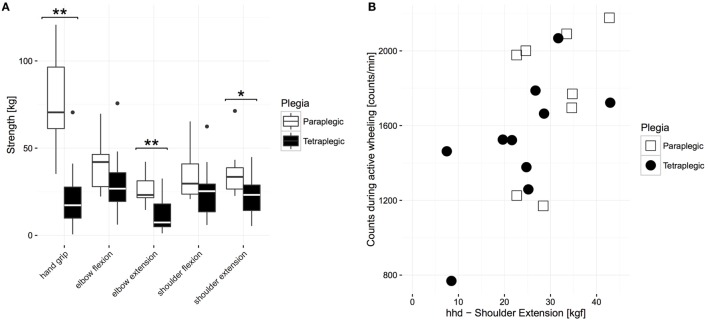
**Comparison of strength values between paraplegic and tetraplegic patients 6 months after injury**. **(A)** The boxplot shows the median of each strength measurement. The bottom represents the first quartile, whereas the top represents the third quartile. The whisker is 1.5 times the interquartile range. Outliers are displayed with points. Significant differences are represented with stars (one star represents alpha ≤ 0.05; two stars represent alpha = 0.01). **(B)** Relationship between AC during active wheeling and HHD scores of shoulder extension. Paraplegic patients are displayed in white or with empty squares, whereas tetraplegic patients are displayed in black or full circles. HHD, hand-held dynamometer.

Next, to determine if the similarity in UL activity between groups was due to similar motor impairments, comparisons between the group means of muscle function were performed. A Mann–Whitney *U* test revealed that proximal MMT scores of paraplegic subjects (median: 40, IQR: 0, mean rank = 20.17) were significantly higher than for tetraplegic subjects (median: 36, IQR: 9.75, mean rank = 10.25, *U* = 28, *z* = −3.29, *p* < 0.01), meaning that the tetraplegic subjects were significantly more impaired than their paraplegic counterparts. As shown in Figure 5, this was also the case for hand strength (mean rank paraplegics = 6.55 and tetraplegics = 16.45, *U* = 6, *z* = −3.58, *p* < 0.001, 11 paraplegics, 11 tetraplegics) and independence in self-care (mean rank paraplegics = 19.83 and tetraplegics = 10.50, median paraplegics = 18, IQR 2, and tetraplegics = 13, IQR: 8; *U* = 32, *z* = −3.011, *p* < 0.001, 12 paraplegics, 16 tetraplegics). However, a further analysis of four key proximal muscles in paraplegic and tetraplegic subjects revealed that the HHD scores of antigravity muscles were equal between paraplegic (mean rank elbow flexors = 17.45, mean rank shoulder flexors = 17.00) and tetraplegic subjects (elbow flexors, mean rank = 11.63, *U* = 50, *z* = −1.87, *p* = 0.06; shoulder flexors, mean rank = 11.94, *U* = 55, *z* = −1.63, *p* = 0.11, Figure 5). This was not the case for elbow extensors (mean rank = 19.73 and 10.06, *U* = 25, *z* = −3.11, *p* < 0.01) and shoulder extensors (mean rank = 18.36 and 11.00, *U* = 40, *z* = −2.37, *p* < 0.05), where the HHD scores were significantly higher in paraplegic subjects compared to tetraplegic subjects (Figure 5). We investigated the relationship of the HHD scores with self-propulsion AC in order to evaluate if impairments in these muscles result in lower AC because the HHD scores of shoulder and elbow extensors were significantly different between the two groups. This was the case for shoulder extensors (*N* = 18, *p* < 0.05, *r* = 0.529, Spearman correlation, Figure 5) but not for elbow extensors (*N* = 18, *p* = 0.28, *r* = 0.267, Spearman correlation).

### Center Differences at 6 Months

**Figure 6:**
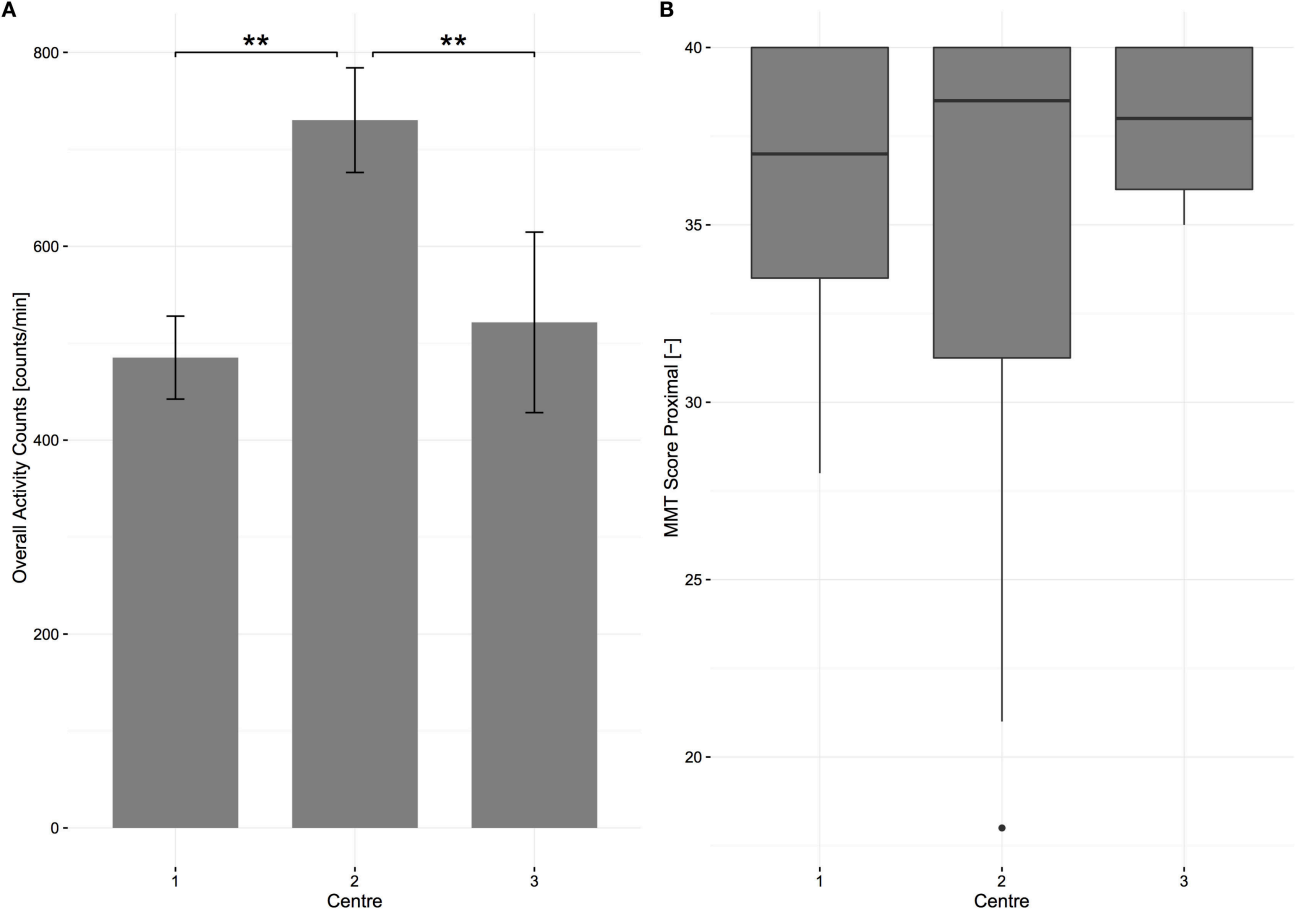
**Center differences in overall activity counts and in scores of proximal muscle strength at 6 months after injury for all patients**. **(A)** The bars represent the means of overall activity counts; error bars represent the 95% confidence interval. Significant differences are represented with stars (two stars equal alpha = 0.01). **(B)** The boxplot shows the median of each strength measurement. The bottom represents the first quartile, whereas the top represents the third quartile. The whisker is 1.5 times the interquartile range. Outliers are displayed with points. MMT, manual muscle testing.

A one-way ANOVA was conducted to determine if overall AC was different for subjects in different centers. Subjects were separated into three groups: center A (*n* = 11), center B (*n* = 12), and center C (*n* = 4). Note that the name of each center is hidden from this analysis in order to guarantee center anonymity. The overall AC was significantly different between the centers *F*(2, 24) = 17.539, *p* < 0.01. The overall AC was highest in center B (730.07 ± 113.68), then center C (521.48 ± 113.20) and lowest in center A (485.12 ± 86.30). Bonferroni *post hoc* analysis revealed that the differences between center A and B [244.94, 95% CI (134.19–355.70)] and between center C and B [208.59, 95% CI (55.40–361.77)] were significant (*p* < 0.01, Figure 6), meaning that subjects in center B were significantly more active. The same analysis was performed for MMT proximal and SCIM self-care in order to determine if this difference between centers was due to differences in muscle impairments or independence. MMT proximal and SCIM self-care were not significantly different between the centers *F*(2, 25) = 0.571 and *F*(2, 25) = 0.847, *p* = 0.572 and *p* = 0.441. Due to the lower number of wheelchair users in center C (three patients), an independent samples *t*-test was conducted to determine if active distance wheeled was different between centers A and B and revealed that the distance wheeled in center A (1682.32 ± 1687.83 m/day, *n* = 7) was not significantly different from center B (2881.77 ± 1001.89 m/day, *n* = 10).

## Discussion

This study assessed changes in UL activity with objective measures of performance at standardized time points during acute rehabilitation. We show that subjects with cervical SCI significantly increase the overall amount of UL activity compared to their thoracic injured counterparts that did not experience significant changes. Moreover, 6 months after injury, subjects with a cervical SCI showed a similar level of UL activity as subjects with a thoracic injury, despite their greater motor impairment. Thus, at this time point post-injury, wearable sensors measure a different level of UL performance as would be predicted by clinical assessments.

Overall AC increased significantly in cervical SCI subjects during the course of acute rehabilitation, suggesting functional recovery of UL movements, which was confirmed by a similar trend in measures of strength and independence. On the contrary, UL activity in paraplegic subjects remained constant confirming that UL motor function is not affected in paraplegic patients, as confirmed by the score of proximal strength. Therefore, in these subjects, inpatient rehabilitative interventions focus on other physical skills (36). Indeed, in this patient group, active peak wheeling velocity increased significantly between 1 and 3 months after injury. This suggests that early rehabilitation focuses on wheelchair training (e.g., improvement of wheelchair handling) in paraplegic subjects compared to tetraplegic subjects. Tetraplegic subjects with high-level injuries are typically not able to propel a manual wheelchair (37), and thus, we did not see a significant improvement in peak wheeling velocity in this group. Our results complement previous findings that showed significantly more time spent on manual wheelchair mobility training for paraplegic subjects, compared to tetraplegic subjects where therapies focused primarily on improving UL function through strengthening and increasing ROM by stretching (37).

In contrast to the overall AC and active peak velocity, there were no significant changes in active distance traveled between the groups. This may be due to the greater unpredictability of global kinematic metrics such as total distance wheeled (34) or due to various confounders, some of which are difficult to control. For example, some subjects (i.e., AIS C or D) progress to functional ambulation as their primary mode of mobility and thus become less dependent on a manual wheelchair (37), and therefore, such subjects most likely decrease their distance wheeled rather than increasing it. Walking detection through wearable sensors is challenging in SCI as ambulation is very heterogeneous in terms of lesions with a broad range of functional impairments that result in several walking alterations (38). Additionally, ambulant SCI subjects use many different assistive devices (e.g., crutches and rollers). For these reasons, algorithms developed for walking detection in other neurological diseases (39–41) have not yet been validated in SCI.

We are aware of only one study that successfully measured distance wheeled in SCI subjects with the help of accelerometers (34). However, all participants were community dwelling, and only two thirds of the enrolled participants were diagnosed with SCI. Additionally, the methods used were not able to differentiate between self-propulsion (active wheeling) or attendant-propulsion (passive wheeling). Therefore, the results of the present study extend the findings for acute SCI by confirming the high variability of global kinematic metrics that fluctuate around 2 km/day and do not change significantly during rehabilitation.

Our results show that there are pronounced inter-subject differences in limb-use laterality within the tetraplegic group, with some tetraplegic subjects showing pronounced limb-laterality soon after injury and others, similarly to paraplegic subjects, not showing any shift in limb-use laterality. Therefore, in order to correctly analyze limb-use laterality, tetraplegic subjects should be split into lateralized and non-lateralized subjects. A powerful method in assisting clinical decision making is the use of *Z*-scores (42). *Z*-scores are the conversion of individual values in terms of SDs from the means by taking into account a reference group. We arbitrarily chose a *Z*-score of 2 as 95.4% of the values fall within 2 SDs from the mean of paraplegic subjects. This is because we have previously shown that paraplegic subjects do not show any limb-use laterality (22) and their limb-use laterality indexes are similar to healthy subjects (43). In analyzing only the lateralized-group, we showed that lateralized cervical subjects significantly decreased limb-use laterality but remained impaired with limb-use laterality values in the same range as a group of chronic tetraplegic subjects who we measured previously (31).

Previously, we have shown that proximal muscle function was strongly related to overall AC during acute inpatient rehabilitation (22). In the present study, we extend these findings and show that this relationship becomes weaker over time. This means that at the beginning of acute rehabilitation, overall UL movements are influenced by the motor impairment of proximal muscles. Therefore, subjects who are more impaired are less active with their ULs. Over time, as patients recover and learn how to perform different tasks through compensatory movement strategies (8), the impairment in some muscles may play a less pronounced role because their function is replaced by other muscles. This is supported by the fact that at 6 months after injury, tetraplegic subjects showed significant differences in muscle impairment, according to the GRASSP MMT, but reached the same level of UL activity (in terms of AC) as paraplegic subjects. Despite the same level of UL activity, the independence score in self-care was significantly different. This might be because, regardless of the ability to perform an activity (e.g., eating with or without a fork with built in cuff), tetraplegic patients are penalized in SCIM scores because they use adaptive devices. Consequently, at the end of the rehabilitation, overall AC may be a better measure of performance compared to clinical assessments. The effect of learning compensatory movement strategies may become obvious by analyzing the change in overall AC compared to the two clinical measures, where the increase in strength and independence seem to stall after 3 months, whereas UL activity keeps increasing.

The outcome measure of overall AC is a purely quantitative measure and does not enable us to evaluate distinct activities. If we split up the overall AC and look more closely into one distinct activity, in this case self-propulsion, we can see a trend toward higher values of self-propulsion AC in paraplegic subjects compared to tetraplegic subjects. Despite this, the difference is small and may not fully reflect the functional impairment of the UL. Therefore, we investigated the motor impairment between para- and tetraplegic subjects in more detail using the HHD. This analysis revealed that, compared to paraplegic subjects, tetraplegic subjects showed no significant difference in the strength of shoulder flexors and elbow flexors, which are muscles that work against gravity (44). The contrary was true for shoulder and elbow extensors. Previously, it has been shown that functional elbow extensors may be crucial for the performance of activities of daily living, including wheelchair propulsion (45). However, although tetraplegic subjects included in our study show a reduction in elbow extensor strength, they do not show a decrease in overall UL activity compared to paraplegic subjects with full elbow extensor function. This indicates that tetraplegic subjects may use other muscles to compensate for the functional deficit in the elbow extensor. It has been suggested that this compensation is mainly driven by scapulothoracic and glenohumeral movements (46) triggered mainly by the shoulder flexors (47). This may suggest that overall AC is directly influenced by these larger anti-gravitation muscles and not by proximal muscles such as the elbow extensors where function can be very well compensated. However, we observed a significant difference between paraplegic and tetraplegic subjects in the shoulder extensor, which is also an anti-gravitation muscle. It has been shown that during ADL, the position of the arms is essentially constrained around the sagittal plane (48) above the waist (49). Therefore, shoulder extensors may not influence ADL, which, as shown in our data, is the main contributor to overall AC. In contrast, during wheelchair propulsion, the shoulder extensor is needed for the recovery phase (50). Our data extend this finding, because ACs during wheeling significantly correlate with HHD score of shoulder extensor.

Furthermore, we aimed to compare UL activity during therapy in contrast to UL activity during leisure time, and we showed that all subjects have a significantly higher UL activity during therapy, whereas the increase was more pronounced in tetraplegic compared to paraplegic subjects. Therefore, we assume that this is due to a major focus on UL therapy in tetraplegic subjects in contrast to paraplegic subjects (37). This may be related to the fact that physical activity levels during inpatient rehabilitation are higher than after discharge (21), suggesting that high levels of UL activity may be confined to therapy time. Interestingly, a recent study demonstrated that this could be successfully counteracted using behavioral interventions that maintain similar physical activity levels after discharge (20). This may be the reason why UL activity during therapy and during leisure time was significantly higher in one rehabilitation center compared to the other two, meaning that this specific center may offer more successful interventions for increasing UL activity. This suggests that an increase in overall UL activity can be achieved by increasing the intensity of existing therapies as well as by offering better opportunities for patients to shape their leisure time in a more physically active manner.

### Limitations

We acknowledge a number of limitations. First, the fact that we see no differences in scores of anti-gravitation muscles between paraplegic and tetraplegic subjects suggests a low stratification of included patients (i.e., low number of patients with high tetraplegia). Second, we could not control for certain cofounders, e.g., the prevalence of ambulatory bouts of mobility, which limits the interpretation of global kinematics metrics (e.g., active wheeling distance).

## Conclusion

This study has shown that tetraplegic subjects significantly improve UL activity during acute rehabilitation, so that by 6 months post-injury, they have reached similar UL activity levels as their paraplegic counterparts. During acute care, sensor-based metrics correlate with UL motor function, whereas this relationship is attenuated later in rehabilitation. This may be due to the task-specific strategies tetraplegic subjects acquire to compensate for deficits in specific UL muscles. Therefore, tracking day-to-day UL activity is crucial to gain valuable insights into the actual impact of a subject’s impairment on their UL movements. Future investigations should focus on controlling for the intensity of activity-based therapies and evaluating their impact on functional recovery and on acquiring reference data to set specific rehabilitation goals. In this way, sensor-based measurements of UL performance may become a powerful tool to tailor rehabilitative therapies to specific subjects.

## Author Contributions

The work presented here was carried out in collaboration between all authors. Contributions statement: conception or design of the work (MB, WP, UA, AB, I-MV, RG, AC, and MS), data acquisition (MB, WP, UA, AB, and I-MV), data analysis (MB, SS, and WP), data interpretation (MB, SS, WP, UA, RG, AC, and MS), drafting or revising critically for important intellectual content (MB, SS, WP, UA, AB, I-MV, RG, AC, and MS), final approval of the version to be published (MB, SS, WP, UA, AB, I-MV, RG, AC, and MS), and agreement to be accountable for all aspects of the work in ensuring the accuracy or integrity of any part of the work (MB, SS, WP, UA, AB, I-MV, RG, AC, and MS).

## Conflict of Interest Statement

The authors declare that the research was conducted in the absence of any commercial or financial relationships that could be construed as a potential conflict of interest.

## Footnotes

^1^www.emsci.org

^2^http://r-project.org
